# Executive Function and Diabetes: A Clinical Neuropsychology Perspective

**DOI:** 10.3389/fpsyg.2020.02112

**Published:** 2020-08-20

**Authors:** Qian Zhao, Yonggang Zhang, Xiaoyang Liao, Weiwen Wang

**Affiliations:** ^1^International Medical Center/Ward of General Practice and National Clinical Research Center for Geriatrics, West China Hospital, Sichuan University, Chengdu, China; ^2^Department of Periodical Press and National Clinical Research Center for Geriatrics, West China Hospital, Sichuan University, Chengdu, China; ^3^Department of Neurology, General Hospital of Western Theater Command, Chengdu, China

**Keywords:** diabetes, executive function, hyperglycemia, hypoglycemia, neuropsychology

## Abstract

**Objective:**

Diabetes is a global public health concern. Management of diabetes depends on successful implementation of strategies to alleviate decline in executive functions (EFs), a characteristic of diabetes progression. In this review, we describe recent research on the relationship between diabetes and EF, summarize the existing evidence, and put forward future research directions and applications.

**Methods:**

Herein, we provide an overview of recent studies, to elucidate the relationship between DM and EF. We identified new screening objectives, management tools, and intervention targets for diabetes management. We also discuss the implications for clinical practice.

**Results:**

In both types 1 and 2 diabetes mellitus (DM), hyperglycemia substantially impairs EF in people of all age groups and ethnicities. Hypoglycemia can similarly impair EF. Interestingly, a decline in EF contributes to DM progression. Glucose dysregulation and EF decline exacerbate each other in a vicious cycle: poor blood glucose control, impaired EF, diabetes management task failure, then back to poor blood glucose control. Many pathophysiological indexes (e.g., obesity, metabolic index, inflammatory and immune factors), neuropsychological indexes (e.g., compliance, eating habits, physical exercise, sleep, and depression), and genetic factors are changed by this pathological interaction between DM and EF. These changes can provide insight into the pathophysiological mechanisms of diabetes-related EF decline.

**Conclusion:**

Further studies, including large-scale prospective and randomized controlled trials, are needed to elucidate the mechanism of the interaction between diabetes and EF and to develop novel strategies for breaking this cycle.

## Introduction

Executive function (EF), an important component of cognitive function, has been the focus of much research in recent years, given its close relationship with chronic non-infectious diseases (Perry et al., 2019). Cognitive function includes all aspects of intellectual and thinking activities, encompassing reasoning, memory, attention, language, and information processing, which are necessary for performing everyday tasks (Huang et al., 2016). EF involves a comprehensive series of advanced cognitive activities that are defined as “one’s ability to plan, initiate, sequence, monitor, and inhibit complex behavior” in the Diagnostic and Statistical Manual of Mental Disorders, 4th ed. (DSM-IV). According to most studies, EFs generally refer to two different levels of cognitive behavior series. On the one hand, EF involve a relatively primary cognitive process, including the effective control of thinking and behavior in the process of achieving goals; on the other hand, EF involve a relatively complex and comprehensive cognitive process, such as task sequencing, problem solving, strategy selection, and so on. Most published studies have focused on the following three specific processes: shifting, working memory, and inhibition. EFs decrease with aging and are impaired in the early stages of dementia and Alzheimer disease (Zhang et al., 2013). EFs are also compromised in chronic diseases such as diabetes. In the decline of EFs with increased age, potential factors such as body mass index (BMI) and intake of processed foods and alcohol are indirectly involved in this process via the cardiovascular system (Cansino et al., 2018; Sakul et al., 2018). Similar factors exist in the interaction between EFs and diabetes.

Diabetes mellitus (DM) is a metabolic disease that is a major public health concern worldwide. The estimated global prevalence rate of diabetes in 2019 was 9.3% (463 million individuals) and is expected to rise to 10.2% (578 million) by 2030 and 10.9% (700 million) by 2045 (Jiang et al., 2019; Kaewput et al., 2019). Development and implementation of diabetes management programs is urgently needed to reduce the DM burden. Diabetes management is closely related to health behavior and can be controlled by self-management, including checking and interpreting blood glucose levels, controlling diet, following an appropriate exercise program, taking medication according to a doctor’s advice, calculating insulin dosage, remembering to carry supplies, and attending regular follow-up appointments. EFs, such as planning and initiating actions, organizing materials, regulating impulses, and shifting attention, are involved in effectively carrying out these behaviors. As DM progresses, EFs can become significantly impaired, which can further exacerbate symptoms. However, the link between EFs and diabetes is unclear. In this review, we discuss the association between EFs and diabetes, including hyperglycemia and hypoglycemia, as well as physiological, behavioral, psychological, and genetic factors.

## Methods

We conducted a search of PubMed to identify relevant studies. Key search terms included the following: (“Diabetes” or “Hyperglycemia” or “Hypoglycemia”) and “Executive function” in the title/abstract (370 articles); (“Diabetes” or “Hyperglycemia” or “Hypoglycemia”) and “Executive dysfunction” in the title/abstract (35 articles).

Based on results of the above two searches, we identified 384 articles by eliminating duplicate and non-English documents.

We grouped the target articles into the following categories: (1) Effect of diabetes on executive function, including diabetes with poor blood glucose control (hyperglycemia, hypoglycemia) and good blood glucose control; (2) Effect of EF level on blood glucose control in patients with diabetes; (3) Factors influencing the relationship between EF and DM. Based on these, we selected 128 articles for further in-depth review.

## Hyperglycemia Leads to EF Impairment

Diabetes is a group of metabolic syndromes characterized by hyperglycemia. Hyperglycemia can cause damage and dysfunction in various tissues, especially the blood vessels, heart, brain, kidneys, eyes, and nerves. People with diabetes are more likely to have lower cognitive function, including EF, a complex function that is necessary for the completion of tasks.

Executive function has been the focus of numerous studies on diabetes. Meta-analyses (12 studies, *n* = 1,784) have demonstrated EF deterioration in patients with type 2 diabetes mellitus (T2DM) as compared with controls (Palta et al., 2014). This relationship between diabetes and EF has been detected in both type 1 diabetes mellitus (T1DM) and T2DM (Dufouil et al., 2015; Musen et al., 2018). However, abnormal blood glucose levels impact EF only after the diagnostic criteria for diabetes are met; there is no obvious effect in the pre-diabetes stage (Geijselaers et al., 2017). Although there are numerous studies on EF in patients with T1DM and T2DM, as yet no studies have been conducted on the relationship between EFs and the other types of diabetes (gestational diabetes and special type diabetes). The impact of diabetes on EF is age-related but can occur at any age. A cross-sectional study among 6,823 older adults showed significantly worse EF performance in patients with T2DM compared with those who did not have diabetes (Mallorqui-Bague et al., 2018). In adolescents, EF is also impaired in T1DM, and the effect of DM on EF becomes more striking as the disease progresses (Luczynski et al., 2019). During aging, the rate of EF decline in patients with diabetes is more rapid (0.022 standard deviations/year) than in those without diabetes (Zheng et al., 2018). Diabetes-related EF impairment is not ethnicity-specific. The Shanghai Aging Study (*n* = 3348) and the Mayo Clinic Study of Aging Study (*n* = 3734), conducted in China and the United States, respectively, had a similar design. Both studies found that DM was associated with worse EF performance (Zhao et al., 2015). Studies conducted in different countries among various ethnic groups have also reached similar conclusions [e.g., Spain (Mallorqui-Bague et al., 2018), Netherlands (Geijselaers et al., 2017), United Kingdom (Zheng et al., 2018), and Poland (Luczynski et al., 2019)].

Numerous studies have been carried out to clarify the association between hyperglycemia and EF. Uncontrolled diabetes (vs. controlled) is associated with lower EF performance, and the decline in EF is largely caused by hyperglycemia [fasting glucose and glycated hemoglobin (HbA1C)], but not insulin resistance (Geijselaers et al., 2017; Luczynski et al., 2019). In a prospective study published in 2020, EF values decreased by 0.058 standard deviations/year for every 1% increase in HbA1c (Yu et al., 2020). Whereas the connection between hyperglycemia and EF decline remain unclear, based on the limited number of studies published to date, we propose that the following pathogenetic mechanisms may underlie this link:

(1) High glucose levels directly induce neuronal apoptosis (Liu Z. et al., 2019).
(2) Diabetes perturbs synaptic plasticity, causing synaptic dysfunction, which might in turn contribute to EF decline (Greenbaum et al., 2014).
(3) Hyperglycemia causes abnormal energy metabolism and neurological dysfunction. Hyperglycemia in diabetes produces nutrient excess in neurons, resulting in abnormal AMPK/PGC-1α (5′-AMP activated protein kinase/peroxisome proliferator-activated receptor γ coactivator-1α) signaling and exhaustion of ATP supply, thereby impairing neural transmission and nerve conduction (Chowdhury et al., 2013). Levels of N-acetylaspartic acid in the right prefrontal cortex are negatively correlated with HbA1c in T2DM (Li et al., 2020), suggesting that blood glucose affects brain metabolite concentrations, which may have an impact on higher neurological activities such as EF.
(4) Microvascular complications caused by hyperglycemia and the resulting neurodegenerative changes impair EF. Magnetic resonance imaging shows that microvascular changes (characterized by a larger white matter lesion volume, cerebral microbleeds, and subcortical infarcts) and neurodegenerative changes (characterized by reduced gray matter) are significantly associated with EF decline in patients with T2DM (Qiu et al., 2014).
(5) Macrovascular complications caused by hyperglycemia impair EF by affecting the blood supply to the brain; hyperglycemia exacerbates this reduction in EF caused by ischemic cerebrovascular disease. *In vivo* studies of patients with T2DM show that obstruction of major cerebral vessels (cortical infarcts) are significantly associated with reduced EF (Qiu et al., 2014). Furthermore, experiments in rats show that hyperglycemia worsens hemorrhagic transformation and neurological deficits after middle cerebral artery occlusion (Hu et al., 2017). This suggests that hyperglycemia is both an initiating and an aggravating factor in the EF decline caused by stroke.
(6) Hyperglycemia causes oxidative stress; activation of aldose reductase, protein kinase C, poly polymerase and cyclooxygenase; as well as endothelial dysfunction, dyslipidemia, and perturbation of calcium balance. The release of cytokines and other factors associated with the inflammatory response act directly on endothelial tissue and neurons, which lead to apoptosis of glial cells and promote the occurrence of cerebral aging (Rojas-Carranza et al., 2018), in turn impairing neurological functioning, including EF.

In summary, hyperglycemia can cause structural, functional, and genetic changes in neurons, which can lead to EF deficits.

## Hypoglycemia Leads to EF Impairment

Hypoglycemia is a common clinical biochemical abnormality in DM treatment. Hypoglycemia results from a perturbed balance between the antidiabetic agent dose, food consumed, and recent exercise. Neurological manifestations of hypoglycemia include reduced consciousness and cognitive dysfunction, convulsions, transient hemiparesis, and stroke. Although many studies have shown that hyperglycemia can lead to EF reduction, low blood glucose level is not preferable. In a recent cohort study of T1DM, significant EF reduction was observed in patients with a history of severe hypoglycemia (Ryan et al., 2016). Even non-severe hypoglycemia may cause deterioration in EF (reduced ability to plan, organize, and make decisions) in T2DM (Nilsson et al., 2019). During hypoglycemia, disorders of energy metabolism, synthesis, and release of neurotransmitters in neurons can result in neuron death (Mohseni, 2014) and functional abnormalities in various areas of the brain. EF, which requires the coordination of multiple brain functional modules, is highly susceptible to hypoglycemia.

## Better EF Enhances Blood Glucose Control

Well-controlled diabetes depends on proper self-management. For example, patients must continuously manage their daily diet and exercise, monitor blood sugar, use medication, and follow up regularly according to their doctor’s instructions. Effective EF is crucial for the long-term implementation of these management measures. Patients with higher EF levels have better dietary compliance and planned blood glucose monitoring (Cuevas and Stuifbergen, 2017). Problems in EF are associated with failures in daily self-regulation (e.g., being distracted or missing blood glucose tests) and with poor adherence and glycemic control (Crochiere et al., 2019). Thus, diabetes can affect EF, and in turn, EF may affect diabetes self-management, which is critical for glycemic control. For health care providers, EF may be a predictor of performance of self-management tasks in patients with diabetes, such that patients with possible self-management disorders can be identified early personalized care plans developed.

A cross-sectional study of 95 African American, American Indian, and white patients with T2DM (average age 72.2 years) found a significant linear correlation between HbA1c and EF. For every 1-point increase in patients’ EF score, HbA1c decreased 0.47% (Nguyen et al., 2010), which suggests that improvement in EF might help patients achieve better glycemic control. This relationship might be owing to the fact that good EF performance can improve patients’ disease-related knowledge and diabetes management behavior. Because both hyperglycemia and hypoglycemia are associated with EF impairment in patients with diabetes, controlling blood glucose to within the standard range is the most important treatment approach for maintaining good EF. Indeed, EF can be improved by controlling blood glucose with oral antidiabetic medications or insulin (Hanyu, 2019). In addition, other treatment methods, including mindfulness meditation, game-based neurofeedback training and exergaming, have been shown to be effective in improving EF. EF training is beginning to make its way into a variety of therapeutic approaches, including diabetes management.

## Factors Involved in Diabetes-Related EF Deficits

A close relationship exists between diabetes and impaired EF. These affect each other in a vicious cycle: poor blood glucose control, impaired EF, diabetes management task failure, then back to poor blood glucose control. However, pathophysiological mechanisms underlying the reduction in EF are unclear. An evaluation of research conducted over the past 20 years suggests that the interaction between diabetes and EF is associated with significant changes in many pathophysiological factors and neuropsychological parameters. These changes may provide clues into the pathophysiological mechanisms underlying diabetes-related EF decline and may also help in identifying early screening markers and therapeutic targets for EF deficits in diabetes.

### Physiological Indexes

In people with diabetes, EF is not only related to blood glucose control but also to various biochemical markers and clinical examination indexes.

#### BMI

It is well known that obesity affects cognitive function. Studies show that EF is the cognitive domain most affected by obesity in both adults (Fitzpatrick et al., 2013) and children (Liang et al., 2014). In patients diagnosed with diabetes, the higher the BMI, the worse the EF performance; even in pre diabetes, BMI is negatively correlated with EF (Bruehl et al., 2010). In addition, the effect of obesity on EF may last for a very long time. A study by Nunley et al. (2015) found that current EF impairment was associated with higher BMI measured 20 years earlier. This suggests that long-term obesity may lead to EF dysfunction in patients with diabetes, and early obesity can partially predict a decrease in EF after 5–20 years. The pathophysiological mechanism between obesity and reduced EF is unclear. According to recent animal studies, adipose-derived stem cells can differentiate into neurons, but obesity inhibits this differentiation and accelerates neuronal apoptosis (Kato et al., 2019). Microvascular lesions and abnormal prefrontal cortex signals can be observed in obese individuals, suggesting that obesity may lead to vascular and neural disorders in the central nervous system (Olver et al., 2018). Therefore, increased BMI in patients with diabetes may affect differentiation of nerve cells and regulation of nerve function via the above pathological mechanisms, which may lead to declining EF.

#### Metabolism Index

Diabetes is characterized by abnormal glucose metabolism, which is mainly manifested by a disorder or deficiency of insulin function. This is also associated with disorders of lipid metabolism and other regulatory hormones. Low serum insulin is associated with EF deficit, suggesting that EF is correlated with serum insulin levels (Han et al., 2015). Elevated high-density lipoprotein cholesterol (HDL-C) levels are significantly associated with better EF performance (Sun et al., 2019), and plasma homocysteine is negatively correlated with EF (Tian et al., 2017). HDL-C is an anti-atherosclerotic lipoprotein that is negatively correlated with cardiovascular risk whereas high homocysteine increases cardiovascular risk. Therefore, disorders of glucose and lipid metabolism in diabetes may affect central nervous system functioning by promoting cerebrovascular disease, which manifests as impairment in EF. It has been found that ghrelin may be an important predictor of EF decline in patients with T2DM (Chen et al., 2017). Ghrelin can stimulate the release of growth hormone from the anterior pituitary and regulate the growth and energy balance of the body, including growth and development of the nervous system. The abnormal increase in ghrelin in diabetes may affect EF by regulating the abnormal proliferation of blood vessels. Serum insulin, HDL-C, homocysteine, and ghrelin may therefore serve as biomarkers or predictors of EF impairment in patients with T2DM, although further research is needed to confirm this conjecture.

#### Inflammation and the Immune Response

Increased cortisol, C-reactive protein, and serum levels of soluble intercellular and vascular adhesion molecules can lead to a decrease in cerebrovascular reactivity and vasodilation function, which may result in EF decline in T2DM (Chung et al., 2015). Inflammation may further impair cerebral vasoregulation, thereby accelerating EF decline and daily activity performance in patients with T2DM. Interestingly, diabetic autoantibodies can induce neurite retraction and cell death in N2A neuroblastoma cells (Zimering, 2017). These observations suggest that activation of inflammatory responses in diabetes may impair central nervous system function by affecting the vasomotor system. The diabetic autoimmune response and inflammation may also directly reduce neuronal viability and disrupt formation of neural connections, which can lead to EF deficits.

### Behavior

#### Eating Habits

Executive function may be related to eating disorders in patients with T1DM. A study of 74 young adults with T1DM showed that reduced EF was associated with significantly greater disordered eating (Broadley et al., 2018). Working memory, planning/organization, shifting, and initiation abilities are specific components of EFs that contribute to variance in eating habits in populations with diabetes. The relationship between EF impairment and poor maintenance of healthy eating habits may lead to common dietary problems among patients with diabetes and may be a target of diabetes prevention or intervention. An intervention study including 1,260 patients found that a healthier baseline diet predicted a favorable change in EF (Beckman et al., 2018). However, the benefits of a healthy diet in EF may vary by ethnicity. In a study among people of different ethnicities, healthy eating behavior in white participants was associated with a lower risk of EF reduction, but no association was observed in black participants (Sundermann et al., 2016). A study in an Israeli population found that use of caffeine may be helpful for EF maintenance in older patients with T2DM, but this relationship could not be confirmed for people of other ethnicities (West et al., 2019).

#### Exercise

Studies show that aerobic exercise programs may slow the progression of neural changes during diabetes and that exercise training improves EFs. In one study, older adults were assigned to exercise five times per week for 3 months on a stationary bicycle. Exercisers with DM exhibited significant gains in EF whereas non-DM exercisers did not (Anderson-Hanley et al., 2012). Hatha yoga can also improve EF in patients with T2DM (Luu and Hall, 2016). Accumulating evidence suggests that physical activity can prevent and delay neuromuscular malnutrition caused by diabetes. Moderate physical activity can improve the ability of neural stem cells to generate neurons and satellite cells to proliferate and differentiate, to fight against neurodegeneration caused by diabetes (Fujimaki et al., 2015). These changes may at least partly underlie the therapeutic effect of exercise on EF in DM. However, studies have not been fully concordant. For example, in one study involving a 6-month multicomponent exercise program, exercise was performed twice a week by patients with T2DM and healthy volunteers; improvements in EF were only observed in the latter group (Coelho Junior et al., 2018). Nonetheless, current research has supported prescribing of exercise by physicians for patients with diabetes, as well as encouraging patients to exercise regularly to help achieve satisfactory diabetes management.

#### Sleep

Sleep is not a “resting state” in which neural activity is absent but rather a process of orderly interaction between various functional modules of the central nervous system. As such, sleep plays a pivotal role in the regulation and maintenance of numerous physiological functions, including EF. A study among 49 children and adolescents with T1DM showed that having normal sleep at night is very important for patients to achieve normal neurological function during the daytime. The disruption of sleep may indicate that children with T1DM will have cognitive impairment and behavioral disorders later in life (Caruso et al., 2014). Timely diagnosis and treatment of sleep disorders could promote EF and help children with T1DM to achieve better blood glucose control. Therefore, assessing sleep status during regular follow-up of children with diabetes may be helpful for early recognition of those who may be at risk of cognitive impairment and behavioral disorders, so as to carry out early treatment interventions. However, for adults with diabetes, longer sleep duration may not be preferable. A study involving 18,769 Swedish adults found that among participants with diabetes, longer sleep duration (≥9 h per day) was associated with poorer EF performance compared with normal sleep duration (7–8 h per day). In contrast, there was no correlation between longer sleep duration and EF in participants who did not have diabetes (Titova et al., 2019). These results suggest that in adults with diabetes, sleepiness may be a symptom of EF decline.

Effective blood glucose control depends on achieving a series of complex target-oriented behaviors, including following the diabetes dietary guidelines, regular physical exercise, adhering to self-monitoring of blood glucose, following a doctor’s advice to use medication, and regular follow-up. Behavioral disorders caused by EF impairment may be one reason that patients with diabetes fail to achieve good diabetes control. Therefore, from the perspective of neuropsychology, maintaining normal EF to ensure that management behaviors are successfully completed may be very important for diabetes self-management. A schematic model of glycemic control can be proposed as follows: EF → self-care behaviors → glycemic control. According to the above causal chain, EF can affect blood glucose control by influencing or promoting self-management behaviors in patients with diabetes, thereby affecting the progress of diabetes.

### Depression

Individuals with diabetes are susceptible to depression via an underlying biological connection. Depression contributes to the link between diabetes and decreased EF. Among the three domains of cognitive function (memory, language, and EF), EF dysfunction exhibits the strongest association with depression (Wei et al., 2019). Moreover, a synergistic effect of depression and diabetes on executive dysfunction has been observed (Wei et al., 2019). Depressed people with diabetes perform significantly worse on EF tasks. However, this correlation was not significant in a subsample of 715 participants taking antidepressants (Guerrero-Berroa et al., 2018), suggesting that depression-mediated impairment in EF among patients with diabetes can be alleviated with antidepressant treatment.

In older community-dwelling adults the United Kingdom, EF performance was diminished in people with diabetes or depression and even worse in those with both (Demakakos et al., 2017). The coexistence of diabetes and depression makes EF decrease more rapidly. In adolescents with T1DM, greater EF dysfunction is independently associated with somatic problems (e.g., aches, pains, nausea, and vision problems) (Crochiere et al., 2019), and EF deficits are associated with lower quality of life, both of which can promote depression. Thus, EF difficulties may predict somatic problems and lower quality of life in individuals with diabetes-related depression.

There is a synergistic relationship among diabetes, EF, and depression, the pathophysiological mechanisms of which are very complex. It has been reported that reduction in the thickness of the prefrontal cortical regions in patients with T1DM contributes to the increased risk of comorbid depression (Lyoo et al., 2012). Higher prefrontal glutamate levels are associated with worse performance in EF as well as with depression (Lyoo et al., 2009). In an animal model of diabetes with depression, some significant pathological features were observed, including neurovascular damage, poor structural integrity of intima, and decreased barrier function. Moreover, apoptosis of neurons increased significantly in a model of comorbidity (Liu J. et al., 2019). Depressed patients with T2DM show decreased connections between functional areas of the brain, mainly the inferior frontal gyrus, cingulate gyrus, central anterior gyrus, and fusiform gyrus; this is associated with reduced performance on neuropsychological tests (Xia et al., 2018). Autoantibodies in peripheral circulation among depressed people with diabetes cause long-lasting depolarization of hippocampal neurons and may result in depression-related adult dentate gyrus neurogenesis via suppression of neurite outgrowth, as well as alteration in membrane excitability (Zimering et al., 2016). DM and depression can cause neuronal apoptosis, cellular metabolic disorder, membrane functional damage, and impaired neural connectivity. These structural and functional perturbations lead to abnormally high neural activity in the brain, resulting in impaired EF. Because depression plays an important synergistic role in the reduction of EF caused by diabetes, the decline in EF may be reversible with drug treatment. Clinicians should pay greater attention to the screening and treatment of depression in patients with DM and closely monitor their EF performance.

### Genetic Factors

Although the correlation between EF and diabetes is found in all age groups, ethnicities, and diabetes types, differences in the interaction between them exist. Research at gene level may help explain for this phenomenon (Table 1). In an Israeli cross-sectional study including 787 patients with T2DM, those with Hp1-1 genotype had poorer EF performance than patients without this genotype (Guerrero-Berroa et al., 2016). However, in another Israeli study among 793 participants, Hp1-1 was not significantly associated with overall EF but only with a specific component of EF, attention/working memory (Guerrero-Berroa et al., 2015). This suggests that patients with T2DM carrying the *HP1-1* gene are more likely to show EF decline than those without this gene, and this difference may be particularly important in the subcomponent of attention/working memory. Patients with T2DM with the short poly-T group of the *TOMM40* gene (rs10524523) exhibited better EF than patients with the very long poly-T group (Greenbaum et al., 2014). Insulin degrading enzyme polymorphism (rs6583817) is associated with EF levels in older adults (McFall et al., 2013). A significant association of the single nucleotide polymorphism rs17518584 with EF performance was found in individuals with T2DM (Greenbaum et al., 2019). Carriers of either one or two copies of the T allele of the *CAMTA1* gene, which is a risk factor for T2DM, perform more poorly in EF tasks than other individuals (Miller et al., 2011). Patients with diabetes who have the apolipoprotein C3 gene (*APOC3*) m482 AA genotype exhibit impaired EF compared with those who have the AG/GG genotype (Smith et al., 2009). Although several genetic phenotypes have been found to be related to the EF deficits caused by diabetes, the underlying pathogenetic mechanisms are far from clear. Further research is needed to elucidate these mechanisms and identify drug targets.

**TABLE 1:** Genes affecting the relationship between DM and EF.

Genotype	Gene located	Studied population	Gene Function	References
variable poly-T (rs10524523)	TOMM40 gene	Jewish elderly with T2DM	Predicts the age of late-onset Alzheimer’s disease	Greenbaum et al., 2014
Hp1-1 phenotype	Hp gene	Ashkenazi vs. non-Ashkenazi Jews older (>65) individuals with T2DM		Guerrero-Berroa et al., 2015; Guerrero-Berroa et al., 2016
SNP within an intron of IDE gene (rs6583817)	Insulin degrading enzyme (IDE) gene	Older adults	Related to late onset Alzheimer’s disease	McFall et al., 2013
SNP of CADM2 gene (rs17518584)	cell adhesion molecule 2 (CADM2) gene	Jewish elderly individuals with T2DM		Greenbaum et al., 2019
T allele of CAMTA1 gene	Calmodulin-binding transcription activator 1 (CAMTA1) gene	Older adults with cardiovascular disease	Risk factor for T2DM	Miller et al., 2011
m482 AA (rs2854117)	Apolipoprotein C3 (APOC3) gene	Hispanics of Caribbean origin subjects with diabetes (aged 45–74)	Related to lipid metabolism	Smith et al., 2009

## Conclusion

Studies on the relationship between EF and diabetes provide insight into the link between the two, although many questions remain. Theoretical models of the relationship between EF and diabetes are not yet fully developed, but we can create a schematic of the relationship between the two in comprehensive analysis of numerous research findings. The connection between diabetes and EF appears to involve complex interactions. Prolonged inadequate self-management of blood glucose levels leads to poor performance in executive tasks. This behavioral impairment in turn further worsens blood glucose control, thereby perpetuating the cycle.

Whereas numerous studies have demonstrated a correlation between EFs and diabetes, these have not clarified the cellular and molecular mechanisms underpinning this relationship, nor has evidence of causality been provided. Identifying whether EFs represent predictors of poor diabetes control or a consequence of diabetes remains an important goal in diabetes research. Furthermore, it is unclear whether all components of EF are equally affected or whether only certain components are selectively perturbed. Further research, including large-scale prospective or randomized controlled trials, are needed to elucidate the pathogenetic mechanisms underlying the connection between DM and EF, to break this cycle.

This review on the relationship between DM and EF and our conclusions are of great relevance in clinical practice. First, the decrease in EF during the course of diabetes is a critical neuropsychological deficit that may lead to poor control of diabetes. Clinicians should pay greater attention to screening of EF in people with diabetes, to identify functional abnormalities in the early stages. Second, maintenance of good EF is critical for the long-term control of diabetes. Greater attention is needed to factors that impact EF, to take measures accordingly and prevent EF decline. Third, EF can be improved through practice and treatment. Given the beneficial effect of good EF for diabetes control, practitioners should promote the treatment and rehabilitation of EF and regard it as a conventional means of improving diabetes management.

## Author Contributions

YZ and XL designed and conceptualized the study. QZ drafted and revised the manuscript. WW revised the manuscript. All authors contributed to the manuscript and approved the final version.

## Conflict of Interest

The authors declare that the research was conducted in the absence of any commercial or financial relationships that could be construed as a potential conflict of interest.
